# Prevalence of musculoskeletal disorders among perioperative nurses: a systematic review and META-analysis

**DOI:** 10.1186/s12891-021-04057-3

**Published:** 2021-02-26

**Authors:** Marco Clari, Alessandro Godono, Giacomo Garzaro, Gianluca Voglino, Maria Rosaria Gualano, Giuseppe Migliaretti, Attilia Gullino, Catalina Ciocan, Valerio Dimonte

**Affiliations:** 1 Department of Public Health and Pediatrics, University of Torino, Via Zuretti 29, 10126 Turin, Italy; 2 Città della Salute e della Scienza di Torino University Hospital, Turin, Italy

**Keywords:** Musculoskeletal diseases, Operating rooms: nurses, Systematic review, Meta-analysis

## Abstract

**Background:**

To evaluate the prevalence of work-related musculoskeletal disorders (WRMSDs) in perioperative nurses and to explore their association with personal characteristics.

**Methods:**

Medline, Cumulative Index to Nursing and Allied Health Literature, Scopus, Web of Science, Cochrane Library and Joanna Briggs Institute Database were systematically searched. A meta-analysis calculating event rates, and relative 95% Confidence Intervals (CI) was performed for each musculoskeletal body region. The contribution of perioperative nurses’ sex, age, and BMI was assessed through a meta-regression.

**Results:**

Twenty-two studies, considering 3590 perioperative nurses, were included in the systematic review. The highest prevalence of WRMSDs was found for the lower-back (62%; 95% CI 0.54–0.70), followed by knee (47%; 95% CI 0.36–0.59), shoulder (44%; 95% CI 0.37–0.51), waist (42%; 95% CI 0.31–0.53), neck (39%; 95% CI 0.29–0.51), ankle-feet (35%; 95% CI 0.22–0.51), upper-back (34%; 95% CI 0.25–0.44), hand-wrist (29%; 95% CI 0.20–0.40), and elbow (18%; 95% CI 0.12–0.26). Meta-regression showed that sex, age, and BMI were not significant predictors of low-back disorders (*p* = 0.69; *R*^2^ = 0).

**Conclusions:**

WRMSDs represent a high prevalence issue among perioperative nurses. Perioperative nurses, in general, are steadily exposed to both physical and temporal risk factors. Further studies should be addressed to identify specific interventions aimed at reducing the burden of WRMSDs including ergonomic education and physical rehabilitation. Our data could be used in future studies as a reference to assess the risk of WRMSDs in other health-care professionals’ population.

**Supplementary Information:**

The online version contains supplementary material available at 10.1186/s12891-021-04057-3.

## Background

Musculoskeletal disorders have been considered as an impactful occupational problem among most working categories [1, 2]. Work-related musculoskeletal disorders (WRMSDs) is an umbrella term for symptoms caused or worsened by work. These disorders are defined as discomfort, impairment, disability or persistent pain in the locomotor system [3].

Furthermore, WRMSDs can be classed as social and economic issues due to their impact on mental and physical health [4]. In fact, they are reported to significantly influence the quality of life, resulting in different degrees of disability, long-term diseases, work restrictions, high treatment costs, absenteeism or even transfers to other jobs [5].

Even if the general population also experience musculoskeletal disorders, some working groups are more encumbered with those diseases. Recent studies have shown that physical factors, such as bending and twisting, manual handling, forceful movements are cardinal determinants of musculoskeletal disorders [2, 6]. From this perspective, it is no surprise that nurses, the largest professional group in health care system, have high incidence rates of musculoskeletal disorders. Nursing has been recognized as a physically demanding work and one of the jobs that continuously face high risks of WRMSDs.

Several studies have focused on the prevalence and risk factors of musculoskeletal disorders among nurses [7, 8] but to the best of our knowledge no meta-analyses were performed. On the other hand, few studies have been conducted internationally among perioperative nurses. In the operating room environment, the nurse’s professional role involves care planning for patients in response to their needs. Working in the operating room carries its own risk of developing musculoskeletal disorders due to the exposure to additional risk factors such as prolonged standing and awkward posture during surgeries.

To our knowledge, no literature review has previously been conducted to determine the occurrence of WRMSDs in this specific population and, accordingly, there is inconsistent evidence on possible interventions to reduce WRMDS in the operating room setting. A better understanding of the real burden of WRMSDs is crucial to highlight this health and safety issue and to promote the implementation of environmental, ergonomic and organizational interventions in these specific working populations.

Thus, the aim of this systematic review and meta-analysis is to evaluate the prevalence of work-related musculoskeletal disorders in perioperative nurses and to explore their association with personal characteristics.

## Methods

Methods of the analysis and inclusion criteria were specified in advance and documented in a protocol, registered on Prospero (https://www.crd.york.ac.uk/PROSPERO; registration number: CRD42019121982). This systematic review was reported following the Preferred Reporting Items for Systematic Reviews and Meta-Analyses (PRISMA) [9]. No ethics approval was needed as all data were obtained from publicly available sources of information.

### Inclusion criteria

- *Population:* perioperative nurses, including operating room nurses, scrub nurses, circulating and anesthesia nurses and perioperative technicians, without age and ethnic restrictions. Since job rotation schedules are often performed in the operating room and they share the same working environment, occupational risk factors between these working categories can be considered overlapping.
- *Exposure*: operating room environment
- *Outcomes:* identify the magnitude and characteristics of WRMSDs in perioperative nurses, define the personal characteristics related to musculoskeletal disorders and evaluate the relationships between the health effects/risk factors and working conditions.

### Exclusion criteria

Articles evaluating exclusively acute musculoskeletal work-related injuries and studies from non-peer reviewed journals will be excluded. Nurses working in home care were not be considered. No limit of publication date was affixed.

### Information sources

Studies were identified by searching electronic databases, scanning reference lists of articles and through consultation with experts in the field. An expert librarian was involved in the search. A systematic search of Medline, Cumulative Index to Nursing and Allied Health Literature (CINAHL), Scopus, Web of Science, Cochrane Library and Joanna Briggs Institute (JBI) Database was conducted from inception to February 2019. A limited update literature search was performed on 31 June 2020. These comprehensive databases were selected because those are broad and extensive in the field of health and nursing sciences. The initial search was applied to Pubmed and then adapted to the other databases.

### Search strategy

We used the following terms to search all database: perioperative nursing, musculoskeletal diseases, occupational diseases, musculoskeletal pain, cumulative trauma disorders. The complete list of the search strings for Pubmed in Online Resource 1.

### Study selection

Following the search, all identified citations were gathered and uploaded on Mendeley Desktop (version 1.19.3; 2008–2018 Mendeley Ltd) and duplicates were removed. Two independent reviewers (MC, AG) screened titles and abstracts for assessment against the inclusion criteria. Afterwards, selected full texts were assessed in detail by two independent reviewers (MC, AG). Any disagreements arisen between the reviewers at any stage of the study selection process were solved through discussion, or with a third reviewer (GG).

### Data collection process and quality appraisal

We developed a data extraction sheet (based on JBI Data Extraction Form for Review for Systematic Reviews and Research Syntheses [10], pilot-tested it on randomly-selected included studies, and refined it accordingly. One review author (AG) extracted the following data (authors, year, country, setting/context, sample size, participants-characteristics/total number, results/findings divided by musculoskeletal body regions, outcome assessed, appraisal, methods of analysis) from included studies and a second author (MC) checked the extracted data. Disagreements were resolved by discussion between the two review authors; if no agreement could be reached, a third author (GG) decided the data to be included. Five authors were contacted for further information. All answered, and one provided numerical data that was only presented graphically in the published paper.

Studies quality was appraised through the Quality Assessment Tool for Observational Cohort and Cross-Sectional Studies by the National Heart, Lung and Blood Institute. Two independent reviewers assessed the quality. Studies could be rated as good, fair or poor-quality basing on the reviewers assessment of risk of bias in the studies due to flaws in study design or implementation.

The level of evidence retrieved were assessed using the Grading Recommendations Assessment, Development and Evaluation (GRADE methodology. We followed GRADE guidelines of evidence about prognosis factors assessing five domains: risk of bias, imprecision, inconsistency, indirectness, and publication bias. The quality of evidence level could be rated from high to very low, depending on the level of confidence that the variation in the risk associated with the prognostic factor lies close to the estimate [11].

### Statistical analysis

Period prevalence, quantified as event rates in 12 months, was the primary measure of WRMSDs occurrence. Proportion meta-analyses were performed by using the statistical software R version 3.6.3, using meta and metafore packages. All the studies presenting comparable outcomes were included. Event rates, and relative 95% Confidence Intervals (CI) were calculated. The Cochran Q and the I^2^ were used to evaluate heterogeneity of studies. In order to tackle potential sources of heterogeneity between studies, the random effects model was used to combine studies if heterogeneity was shown (Cochran Q *p* < 0.10 and I^2^ > 50%) [12]. Moreover, to assess whether or not the publication bias was present, statistical analyses and graphs representing funnel plots were performed. Lastly, to examine the contribution of perioperative nurses’ personal characteristics (sex, age, and BMI) to the heterogeneity in study findings, a meta-regression was performed. *P*-values < 0.05 were considered statistically significant.

## Results

**Fig. 1:**
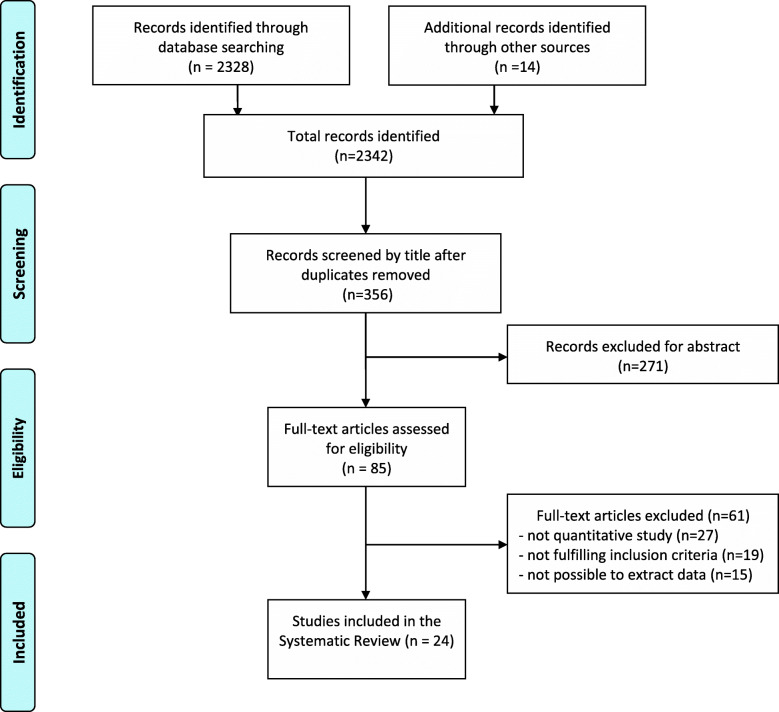
Literature review flow-diagram

The literature research yielded a total of 2328 citations. Fourteen additional citations were added by checking the references of relevant papers and hand-searching for studies that have cited these papers. After adjusting for duplicates and screening by title, 356 articles remained. Of these, 271 studies were discarded after reviewing the abstracts. The full text of the remaining 85 citations was examined in detail. Then, 61 studies were excluded as described: 27 were not quantitative studies, 19 did not fulfill the inclusion criteria, and 15 had no pertinent data to extract. Finally, a total of 24 studies were identified for inclusion in the systematic review (Fig. 1) [13–36].

The studies were published from 2003 to 2019. They all used a cross-sectional design except from Bakola et al. [30] that used a prospective design and Keriri et al. [21] that added a nested case control study to the cross-sectional design. The total sample of perioperative nurses included was 3590; most were female (77.2%), with a mean age of 37.6 years.

Mean seniority, calculated as years working as a perioperative nurse, was 11.3 years, working on average 7.8 h/day. Most of the studies participants had a normal BMI (range: 22.8–26.9).

To evaluate the prevalence of WRMSDs, ten studies [13, 17, 19–21, 24, 28, 30, 32, 35] used Research-Made Questionnaire (R-M Q), nine [14, 15, 18, 22, 26, 27, 29, 31, 36] Nordic Musculoskeletal Questionnaire (NMQ), two [29, 36] Rapid Entire Body Assessment (REBA), one [16] Musculoskeletal Symptoms Survey (MSS), one [23] American National Standards Institute Z-365 (ANSI Z-365), one [25] North American Spine Society-Questionnaire (NAAS-Q), one [34] Modify Oswestry Low Back Pain Disability Questionnaire (MOLBPDQ), one [33] Disabilities of the Arm, Shoulder and Hand (DASH). Moreover, two studies [26, 27] added a clinical examination to define the magnitude of WRMSDs.

Few studies evaluated the duration and intensity of symptoms, indicating a high prevalence of long-lasting symptoms especially for low-back pain [20, 21, 26, 30]. A significant association between WRMSDs and perioperative nurses’ personal characteristics was reported in five studies for the female sex [18, 21, 22, 29, 33] and age [17, 18, 20, 26, 29], in four studies for BMI [18, 26, 28, 29], in two studies for seniority [26, 31] and number of working hours [22, 33]. Several studies described the postures and movements of PNs [15, 16, 19, 26], but the association with WRMSDs reported conflicting results [14, 18, 21, 28, 30, 32, 34, 36].

All the studies had a fair quality rating. The complete critical appraisal is reported in Online Resource 2.

A detailed summary of the characteristics of the included studies is reported in Table 1.

**Table 1:** Characteristics of the included studies

Author/Year	Country	Study design	Sample size/sex/age	BMI	Data assesment	Critical appraisal
Ruzafa-Martinez et al. (2003) [13]	Spain	cross-sectional	24 PNs: 22 F; 2 M	^a^	R-M Q	fair
Bos et al. (2007) [14]	Netherlands	cross-sectional	381 PNs: 324 F; 57 M. Age: 40 ± 10	24 ± 4	NMQ	fair
Meijsen et al. (2007) [15]	Netherlands	cross-sectional	463 PNs: 394 F; 69 M. Age: 36 ± 10.3	^a^	NMQ	fair
Sheikhzadeh et al. (2009) [16]	US	cross-sectional	32 PNs. Age: 43.9 ± 9.1	^a^	MSS	fair
Choobineh et al. (2010) [18]	Iran	cross-sectional	375 PNs: 249 F; 126 M. Age: 31.5 ± 8.5	22.8 ± 3.3	NMQ	fair
Moscato et al. (2010) [17]	Italy	cross-sectional	185 PNs: 73 F; 112 M. Age: 36.1 ± 7.1	M 22.6 ± 3.2 F 25.5 ± 3.7	R-M Q	fair
Aljeesh et al. (2011) [19]	Palestine	cross-sectional	143 PNs: 33 F, 110 M. Age: 33.7 ± 9.59	26.6 ± 4.5	R-M Q	fair
Hinmikaiye et al. (2012) [20]	Nigeria	cross-sectional	80 PNs: 56 F; 24 M.	^a^	R-M Q	fair
Simonsen et al. (2012) [32]	Sweden	cross-sectional	99 PNs	^a^	R-M Q	fair
Keriri et al. (2013) [21]	Saudi Arabia	cross-sectional + nested case control	126 PNs (94 ORNs, 32 Technicians): 99 F; 27 M. Age: 34.0 ± 8.0	24.9 ± 4.5	R-M Q	fair
Arsalani et al. (2014) [22]	Iran	cross-sectional	117 PNs	^a^	NMQ	fair
Ryu et al. (2014) [23]	South Korea	cross-sectional	35 PNs: 35 F	^a^	ANSI Z-365	fair
Nützi et al. (2015) [25]	Switzerland	cross-sectional	116 PNs: 97 F; 19 M. Age: 3.9 ± 11.9	^a^	NAAS-Q	fair
Uğurlu et al. (2015) [24]	Turkey	cross-sectional	74 PNs: 46 F; 28 M. Age: 29.3 ± 6.7	^a^	R-M Q	fair
Arvidsson et al. (2016) [27]	Sweden	cross-sectional	305 PNs: 305 F. Age: 47 ± 10	24 ± 4	CE + NMQ	fair
Asadi et al. (2016) [28]	Iran	cross-sectional	45 PNs	^a^	R-M Q	fair
El Ata et al. (2016) [26]	Egypt	cross-sectional	184 PNs: 155 F; 29 M. Age: 20–50 ys	< 30	CE + NMQ	fair
Homaid et al. (2016) [35]	Saudi Arabia	cross-sectional	41 PNs (34 ORN, 7 Technicians)	^a^	R-M Q	?
Bakola et al. (2017) [30]	Greece	prospective	44 PNs: 35 F; 9 M. Age: 42.7 ± 5.5	24.7 ± 4.3	R-M Q	fair
Mahmoudifar et al. (2017) [29]	Iran	cross-sectional	50 PNs	^a^	NMQ + REBA	fair
Nasiri-Ziba et al. (2017) [31]	Iran	cross-sectional	133 PNs: 103 F; 30 M. Age: 29.1 ± 6.8	23.1 ± 2.7	NMQ	fair
Jeyakumar et al. (2018) [34]	US	cross-sectional	250 PNs: 220 F; 30 M	24.5	MOLBPDQ	fair
Asghari et al. (2019) [36]	Iran	cross-sectional	144 PNss: 115 F; 29 M. Age: 34.6 ± 6.6	24.4 ± 2.9	NMQ + REBA	?
Clari et al. (2019) [33]	Italy	cross-sectional	144 PNs: 114 F; 30 M	^a^	DASH	fair

^a^data not available

*BMI* Body Mass Index, *M* Male, *F* Female, *PNs* perioperative nurses, *R-M Q* Research-Made Questionnaire, *NMQ* Nordic Musculoskeletal Questionnaire, *MSS* Musculoskeletal symptom Survey, *ANSI Z-365* American National Standards Institute Z-365, *NAAS-Q* North American Spine Society-Questionnaire, *CE* Clinical Examination, *REBA* Rapid Entire Body Assessment, *MOLBPDQ* Modify Oswestry Low Back Pain Disability Questionnaire, *DASH* Disabilities of the Arm, Shoulder and Hand

### Meta-analysis

Table 2 shows the 12 months prevalence of WRMSDs in the identified 9 musculoskeletal body regions.

**Table 2:** Twelve-month prevalence of WRMSDs in musculoskeletal body regions, certainty assessment and level of evidence

№ of studies	Certainty assessment						Effect		Certainty
	Study design	Risk of bias	Inconsistency	Indirectness	Imprecision	Other considerations	№ of individuals	Event rate(95% CI)	
***Neck*** (follow up: 12 months; assessed with: Prevalence)									
11	observational studies	not serious ^a^	serious ^b^	not serious ^c^	serious ^d^	publication bias strongly suspected ^e^	1900	39% (29–51)	⨁◯◯◯VERY LOW
***Shoulder*** (follow up: 12 months; assessed with: Prevalence)									
10	observational studies	not serious ^a^	serious ^b^	not serious ^c^	not serious ^f^	publication bias strongly suspected ^e^	1518	44% (37–51)	⨁⨁◯◯LOW
***Elbow*** (follow up: 12 months; assessed with: Prevalence)									
8	observational studies	*not* serious ^a^	serious ^b^	not serious ^c^	not serious ^f^	publication bias strongly suspected ^e^	1102	18% (12–26)	⨁⨁◯◯LOW
***Hand-wrist*** (follow up: 12 months; assessed with: Prevalence)									
10	observational studies	not serious ^a^	serious ^b^	not serious ^c^	not serious ^f^	publication bias strongly suspected ^e^	1518	29% (20–40)	⨁⨁◯◯LOW
***Upper-back*** (follow up: 12 months; assessed with: Prevalence)									
8	observational studies	not serious ^a^	serious ^b^	not serious ^c^	not serious ^f^	publication bias strongly suspected ^e^	994	34% (25–44)	⨁⨁◯◯LOW
***Lower-back*** (follow up: 12 months; assessed with: Prevalence)									
19	observational studies	not serious ^a^	serious ^b^	not serious ^c^	not serious ^f^	publication bias strongly suspected ^e^	3139	62% (54–70)	⨁⨁◯◯LOW
***Waist*** (follow up: 12 months; assessed with: Prevalence)									
7	observational studies	not serious ^a^	serious ^b^	not serious ^c^	not serious ^d^	publication bias strongly suspected ^e^	1020	42% (31–53)	⨁⨁◯◯LOW
***Knee*** (follow up: 12 months; assessed with: Prevalence)									
8	observational studies	not serious ^a^	serious ^b^	not serious ^c^	not serious ^f^	publication bias strongly suspected ^e^	1070	47% (36–59)	⨁⨁◯◯LOW
***Ankle-feet*** (follow up: 12 months; assessed with: Prevalence)									
9	observational studies	not serious ^a^	serious ^b^	not serious ^c^	serious ^d^	publication bias strongly suspected ^e^	1375	35 (22–51)	⨁◯◯◯VERY LOW

^a^Studies have a fair quality rating

^b^I2 > 50%

^c^The studied population correspond to the population in study

^d^The effect on clinical action could differ depending on the 95% CI

^e^Funnel and doi plot reporting major asymmetry

^f^The effect on clinical action not differ depending on the 95% CI

**Fig. 2:**
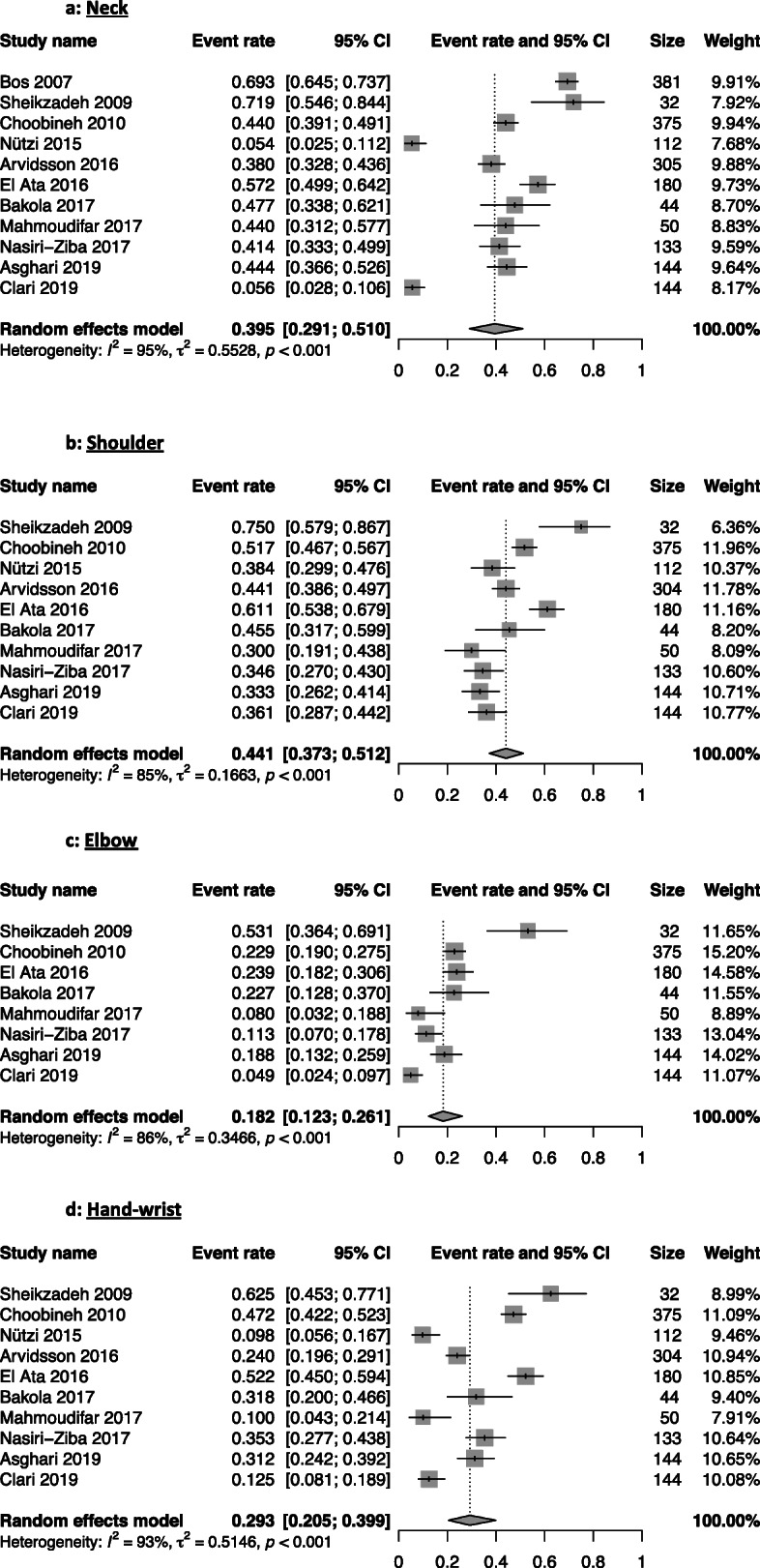
Meta-analysis of the prevalence of upper-limbs work-related musculoskeletal disorders for the neck (**a**), shoulder (**b**), elbow (**c**), and hand-wrist (**d**) musculoskeletal body regions

**Fig. 3:**
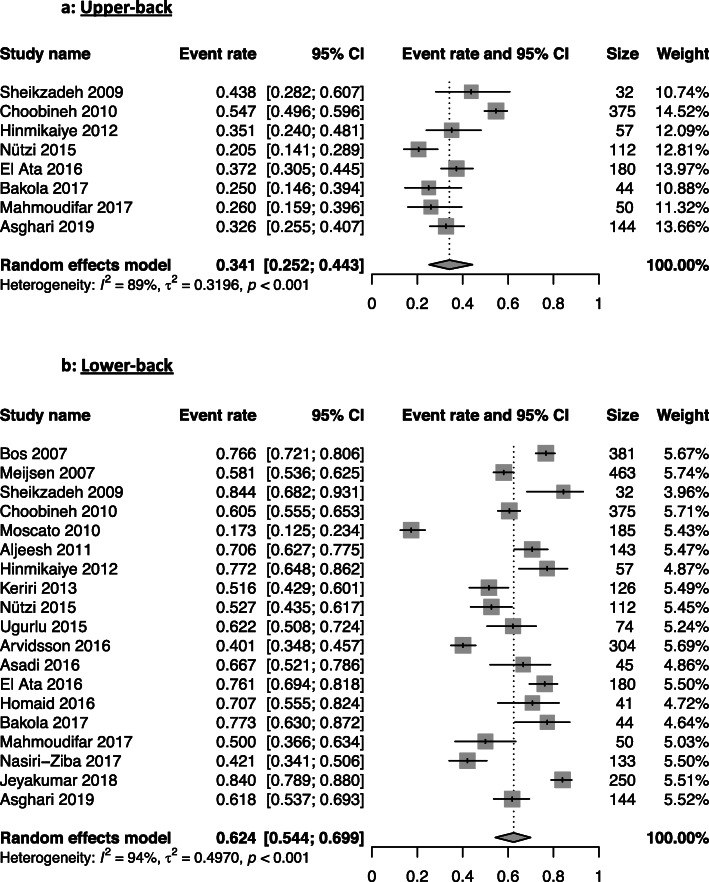
Meta-analysis of the prevalence of back work-related musculoskeletal disorders for the upper-back (**a**), and lower-back (**b**) musculoskeletal body regions

**Fig. 4:**
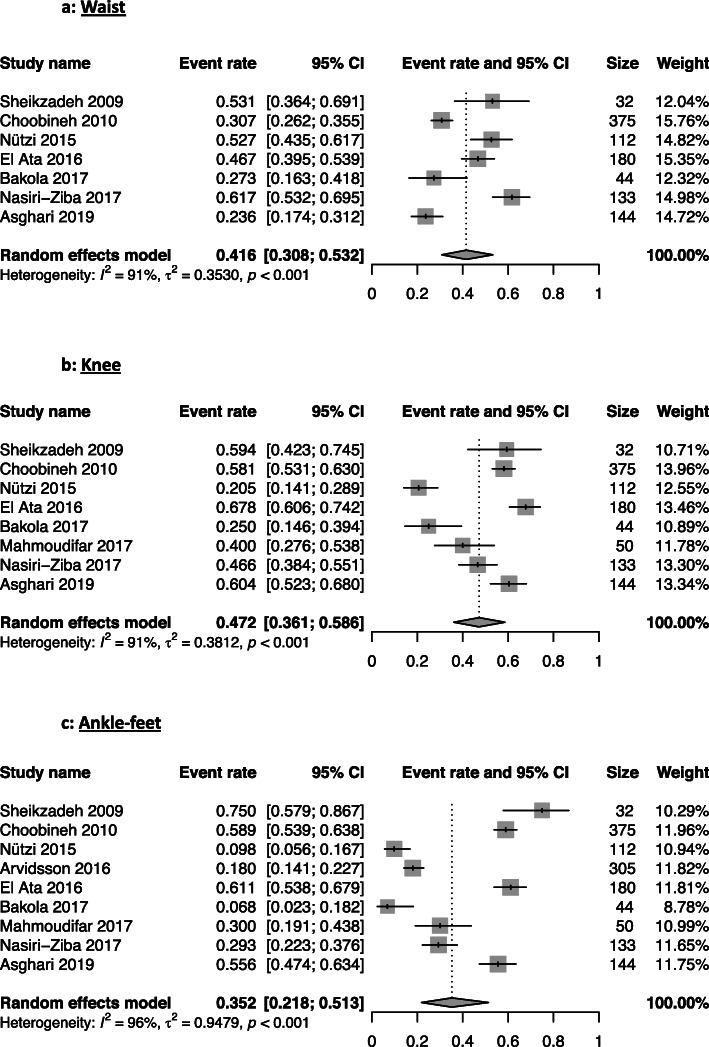
Meta-analysis of the prevalence of lower-limbs work-related musculoskeletal disorders for the waist (**a**), knee (**b**), and ankle-feet (**c**) musculoskeletal body regions

Lower back issues were the most present WRMSD with a 62% prevalence from 19 studies [14–21, 24–31, 34–36]. The knee region had a WRMSDs prevalence of 47%, followed by the shoulder (44%), the waist (42%) regions. The other regions had the following prevalence: neck (39%), upper-back (34%), ankle-feet (35%), hand-wrist (29%), and elbow (18%) (Table 2). The forest plots illustrate the meta-analyses of the nine musculoskeletal body regions, grouped into upper-limbs (Fig. 2), back (Fig. 3), and lower-limbs (Fig. 4).

There was evidence of significant heterogeneity (I^2^ > 50%) in all the meta-analyses performed. The funnel plots for all the meta-analyses were scattered and asymmetrical, representing a possible presence of reporting bias.

Due to the limited number of studies considering perioperative nurses characteristics, it was possible to perform a meta-regression for lower back region only. This meta-regression showed that sex, age, and BMI were not significant predictors of low back disorders (*p* = 0.69; *R*^2^ = 0).

### Level of evidence

The overall quality of evidence was low for all the body regions, but for the neck and ankle-feet regions the quality of evidence was very low. There is limited certainty that the variation in risk is associated with the perioperative nurse job. The level of evidence for observational studies was downgraded due to the high heterogeneity of the pooled meta-analyses and the suspected publication bias highlighted by the major asymmetry of the funnel and doi plots. The estimates with low quality of evidence were downgraded due to the large 95% CI.

## Discussion

This systematic review and meta-analysis evaluated the prevalence of WRMSDs in perioperative nurses and their association with personal characteristics. Musculoskeletal disorders are one of the highest contributors to global disability [37]. Recently, the World Health Organization estimated that between 20 and 33% of general population live with a painful musculoskeletal condition [38]. In particular, WRMSDs remain the most common work-related health problem in the European Union and workers in all sectors and occupations can be affected. Of all workers in the European Union with a work-related health problem, 60% identify musculoskeletal disorders as their most serious issue [6].

Specifically, health-care professionals might be at high risk of incurring in musculoskeletal disorders [39]. Our results are in line with literature for other health-care professionals. According to a recent systematic review, nearly three out of four nurses employed in a hospital suffered from pain or discomfort in at least one of any of the musculoskeletal body regions during the past 12 months of work [5]. In this review, the three musculoskeletal body regions mostly affected were: lower back (65.3%), knees (56.2%) and neck (49.8%) [5]. Also, results from a cross-sectional study conducted on nursing aides working in nursing homes showed that 87.4% of the study population experienced musculoskeletal disorders in the previous year (lower back 41.4%, shoulders 53%, knees 37.5%) [40]. Furthermore, a high prevalence of WRMSD has been also observed in X-ray technologists with an overall 12-months prevalence of low back pain of 75.1% and a 64.2% of the neck-shoulder segment [14]. In particular, the operating room setting appears to be at high risk of causing WRMSDs. Epstein et al. reported, among a large sample of surgeons and interventionalists, an overall 12-month prevalence of neck pain of 60%, of shoulder pain of 52%, of back pain of 49% and of upper extremities of 35% [41].

The highest prevalence of musculoskeletal disorders in the working population is attributable to disorders at the back region. Consistently, the general population shows a lower back pain life-time prevalence between 51 to 90% [42]. Just for the low back region, it has been estimated that approximately $50 billion per year is spent in the United States [43]. Nursing has been identified amongst the top professions at risk of lower back pain [44]. Our results showed that more than 60% of perioperative nurses suffered from work-related lower back pain, and this is particularly relevant if we consider that perioperative nurses, in general, could be highly exposed to both physical and temporal risk factors, such as low temperature, highly repetitive tasks at high force, and frequent use of vibrating instruments. Furthermore, perioperative health-care professionals have to maintain static postures during surgical procedures for an extended time [45]. The impossibility of switching body positions is a relevant contributor to fatigue and health problems related to the lower back region [46].

Several personal characteristics could be related to WRMSDs. Among these characteristics, the female sex seems to be associated with a greater risk of lower-back problems both in nurses [47, 48] and in the population of operating room nurses [18, 21, 22, 25, 29, 33]. Despite this, in our review female sex was not a significant predictor of low back disorders. Traditionally, sex has not been considered a predictor of WRMSDs, but a confounding or modifying factor due to the mixed exposure to work and extra-work activities. However, according to some recent studies, employed women seem to have an increased risk of WRMSDs, in particular in the upper-body musculoskeletal region. The most likely explanation of the increased risk of WRMSDs in female workers might be the differences in somatic, hormonal, and psychological aspects. Furthermore, women are more prone to WRMSDs in cold working environments [49] and there can be differences in repetitive procedures used between males and females [50]. Moreover, women are usually more in charge of the domestic work, and this further burden could increase musculoskeletal issues [51]. The combined work-home exposure to musculoskeletal demands could also reduce the opportunity for recovery time, and for strengthening body muscles with a higher risks of overweight consequences [52]. Lastly, future studies need to understand the links between biological and psychosocial aspects addressing not only the somatic and functional differences between male and female sex but also accounting for the similarities in male and female behaviors [53].

Percentages of overweight and obesity are high among employed adults with rising rates over the past few decades [54]. Several studies have linked a high BMI with musculoskeletal disorders and the repetitive work [55, 56]. This statement could have been true especially for our population, particularly those exposed to prolonged repetitive tasks in awkward postures. Surprisingly, although some studies [18, 26, 28, 29] considered in our meta-analysis reported an association between an increased BMI and WRMDs, the meta-regression results did not confirm this assumption. This might be due to a younger age of perioperative nurses compared to other nursing roles [57], and that the BMI alone could not represent a reliable predictor.

It is also known that musculoskeletal disorders related to work are a major cause of disability in older workers [58]. In this regard, more than one third of the nursing workforce in the United States is between the ages of 50 and 64 [59]. In our sample, the mean age was lower with an average age of 36.7 years. This could be explained in part by the fact that perioperative nurses usually begin their career right after the graduation, and that through the years they usually change their position from the operation room to outpatients’ settings, usually with minor physical burden. This assumption could explain the absence of association in the meta-regression. Only a few studies [17, 18, 20, 28, 29] have shown a correlation between age and WRMSDs.

To date, scientific literature regarding possible interventions to reduce WRMDS in the operating room setting is poor. A multidisciplinary approach that takes into consideration environmental, ergonomic, and organizational factors would be recommended to address this issue. In this regard, particular attention should be given to the evaluation of repetitive motions and prolonged restricted posture, handling heavy weight, forceful gripping, low temperatures, the use of vibrating instruments and to the frequency, intensity, and duration of each task performed at work. Possible ergonomic interventions to minimize risks and reduce the incidence of work-related lower back disorders should include: propping alternating feet on foot stools, using anti-fatigue mats, using sit/stand stools, limiting standing times, wearing appropriate footwear, and implementing postural exercises such as regular contraction and relaxation of muscles during the surgical procedures [60]. Moreover, perioperative nurses could benefit from ergonomic education and physical rehabilitation, if needed. Also, organizational strategies can be adopted to allow a more effective management of human resources, especially when assigning workers to specific jobs or tasks such as job mechanization, job rotation, job enlargement, and the design of a safe work environment [61]. Due to the multifaceted nature of WRMDSs and the complexity of the perioperative nurse job, the proposed preventive strategies could be most beneficial if combined. For these reasons, future efforts should be directed to assess the real effectiveness of preventive measures and to standardize their implementation.

This review has some potential limitations. Data from the articles included in the meta-analysis may not represent the general population heterogeneously, in fact about a quarter of the studies were conducted in Iran, limiting the generalizability due to contextual factors. The high heterogeneity in the meta-analyses could be related to several elements. Firstly, the clinical settings and the role and responsibilities of perioperative nurses could differ between countries. Furthermore, it was not possible to stratify by surgical specialties due to the lack of data and even within the same surgical specialty, the surgical procedures could differ for the adoption of specific surgical techniques. Moreover, the diagnosis of WRMSDs is quite difficult itself, including both clinical-diagnostic heterogeneity and subjective psychosocial components. In this regard, most included studies evaluate the prevalence of WRMSDs through self-reported measures without imaging support nor clinical examination. Lastly, the lack of data from included studies could have limited the results of the meta-regression. Despite these limitations this is the first systematic review conducted on this topic providing a meta-analysis.

## Conclusions

WRMSDs represent a high prevalence issue among perioperative nurses. The musculoskeletal body regions mostly affected were lower back, shoulder, waist, and knee. Age, sex and BMI seem not to be related to WRMSDs prevalence. Environmental, ergonomic and organizational factors should be implemented trying to reduce the burden of WRMSDs in perioperative nurses. Our data could be used in future studies as a reference to assess the risk of WRMSDs in other health-care professionals’ population.

## Supplementary Information

**Additional file 1.** Search strings for Pubmed.

**Additional file 2. **Quality Assessment.

## Data Availability

The majority of data generated or analysed during this study are included in this published article [and its supplementary information files]. Further datasets used and/or analysed during the current study are available from the corresponding author on reasonable request.

## Abbreviations

WRMSDs: Work-related musculoskeletal disorders
CI: Confidence Intervals
PRISMA: Preferred Reporting Items for Systematic Reviews and Meta-Analyses
CINAHL: Cumulative Index to Nursing and Allied Health Literature
BMI: Body Mass Index
PNs: Perioperative nurses
R-M Q: Research-Made Questionnaire
NMQ: Nordic Musculoskeletal Questionnaire
MSS: Musculoskeletal symptom Survey
ANSI Z-365: American National Standards Institute Z-365
NAAS-Q: North American Spine Society-Questionnaire
CE: Clinical Examination
REBA: Rapid Entire Body Assessment
MOLBPDQ: Modify Oswestry Low Back Pain Disability Questionnaire
DASH: Disabilities of the Arm Shoulder and Hand
