# Supplementary material for: Prevalence of musculoskeletal disorders among perioperative nurses: a systematic review and META-analysis
Source: BMC Musculoskelet Disord. 2021 Feb 26;22:226. doi: 10.1186/s12891-021-04057-3 (PMC7908783; doi:10.1186/s12891-021-04057-3)
Supplement: Supplementary file 1 — Additional file 1. Search strings for Pubmed. [file 12891_2021_4057_MOESM1_ESM.docx]

**Appendix 1** – Search strings for Pubmed

- ("Perioperative Nursing"[Mesh] OR operating-room-nurs* OR theatre-nurs* OR scrub-nurs* OR surgical-nurs*) AND ("Musculoskeletal Diseases"[Mesh] OR bone-diseas* OR cartilage-diseas* OR fasciitis OR foot-deformit* OR foot-diseas* OR hand-deformit* OR jaw-diseas* OR joint-diseas* OR muscular-diseas* OR musculoskeletal-diseas* OR musculoskeletal-abnormalit* OR rheumatic-diseas*)
- ("Perioperative Nursing"[Mesh] OR operating-room-nurs* OR theatre-nurs* OR scrub-nurs* OR surgical-nurs*) AND ("Occupational Diseases"[Mesh] OR occupational-diseas* OR occupational-illnes*)
- ("Perioperative Nursing"[Mesh] OR operating-room-nurs* OR theatre-nurs* OR scrub-nurs* OR surgical-nurs*) AND ("Back Pain"[Mesh] OR "Low Back Pain"[Mesh] OR "Musculoskeletal Pain"[Mesh] OR "Chronic Pain"[Mesh] OR "Shoulder Pain"[Mesh] OR "Neck Pain"[Mesh] OR "Pelvic Pain"[Mesh] OR "Cumulative Trauma Disorders"[Mesh] OR Musculoskeletal-injur*)
- ("Perioperative Nursing"[Mesh] OR operating-room-nurs* OR theatre-nurs* OR scrub-nurs* OR surgical-nurs*) AND (UL-WMSDS OR ORN OR PMFR OR ergonomic* OR LBP)
