# Supplementary material for: Prevalence of musculoskeletal disorders among perioperative nurses: a systematic review and META-analysis
Source: BMC Musculoskelet Disord. 2021 Feb 26;22:226. doi: 10.1186/s12891-021-04057-3 (PMC7908783; doi:10.1186/s12891-021-04057-3)
Supplement: Supplementary file 2 — Additional file 2. Quality Assessment. [file 12891_2021_4057_MOESM2_ESM.docx]

| **Author** | **Year** | **1** | **2** | **3** | **4** | **5** | **6** | **7** | **8** | **9** | **10** | **11** | **12** | **13** | **14** |
| --- | --- | --- | --- | --- | --- | --- | --- | --- | --- | --- | --- | --- | --- | --- | --- |
| Ruzafa- Martinez *et al.* | 2003 | yes | yes | yes | yes | no | no | no | yes | yes | no | no | NA | NA | no |
| Bos *et al.* | 2007 | yes | yes | yes | yes | no | no | no | yes | yes | no | yes | NA | NA | no |
| Meijsen *et al.* | 2007 | yes | yes | yes | yes | no | no | no | no | yes | no | yes | NA | NA | no |
| Sheikhzadeh *et al.* | 2009 | yes | yes | yes | yes | no | no | no | no | yes | no | yes | NA | NA | no |
| Choobineh *et al.* | 2010 | yes | yes | yes | yes | no | no | no | yes | yes | no | yes | NA | NA | no |
| Moscato *et al.* | 2010 | yes | yes | yes | yes | no | no | no | no | no | no | yes | NA | NA | no |
| Aljeesh *et al.* | 2011 | yes | yes | yes | yes | no | no | no | yes | no | no | yes | NA | NA | no |
| Hinmikaiye *et al.* | 2012 | yes | yes | yes | yes | no | no | no | no | no | no | yes | NA | NA | no |
| Keriri HM *et al.* | 2013 | yes | yes | yes | yes | no | no | no | yes | no | no | yes | NA | NA | no |
| Arsalani *et al.* | 2014 | yes | yes | yes | yes | no | no | no | no | no | no | yes | NA | NA | no |
| Ryu *et al.* | 2014 | yes | yes | yes | yes | no | no | no | no | no | no | yes | NA | NA | yes |
| Nützi *et al.* | 2015 | yes | yes | yes | yes | no | no | no | no | no | no | yes | NA | NA | no |
| Uğurlu *et al.* | 2015 | yes | yes | yes | yes | no | no | no | yes | no | no | yes | NA | NA | no |
| Arvidsson *et al.* | 2016 | yes | yes | yes | yes | no | no | no | no | yes | no | yes | NA | NA | no |
| Asadi P *et al.* | 2016 | yes | yes | yes | yes | no | no | no | no | no | no | yes | NA | NA | no |
| Homaid *et al.* | 2016 | yes | yes | yes | yes | no | no | no | yes | no | no | yes | NA | NA | no |
| El Ata *et al.* | 2016 | yes | yes | yes | yes | no | no | no | yes | no | no | yes | NA | NA | no |
| Bakola *et al.* | 2017 | yes | yes | yes | yes | no | no | no | yes | no | no | no | NA | NA | no |
| Mahmoudifar *et al.* | 2017 | yes | yes | yes | yes | no | no | no | yes | no | no | yes | NA | NA | no |
| Nasiri-Ziba *et al.* | 2017 | yes | yes | yes | yes | no | no | no | yes | yes | no | yes | NA | NA | no |
| Simonsen *et al.* | 2017 | yes | yes | yes | yes | no | no | no | no | no | no | yes | NA | NA | no |
| Jeyakumar  *et al.* | 2018 | yes | yes | yes | yes | no | no | no | yes | no | no | yes | NA | NA | no |
| Asghari *et al.* | 2019 | yes | yes | yes | yes | no | no | no | yes | yes | no | yes | NA | NA | no |
| Clari *et al.* | 2020 | yes | yes | yes | yes | yes | no | no | yes | yes | no | yes | NA | NA | no |
